# Longitudinal Impact of Transition to Caregiving on Cognitive Functioning: A Matched Case-Control Study

**DOI:** 10.1093/geroni/igaf122.2980

**Published:** 2025-12-31

**Authors:** Joanne Elayoubi, William Haley, Monica Walters, Michael Crowe, Gizem Hueluer

**Affiliations:** University of South Florida, Tampa, Florida, United States; University of South Florida, University of South Florida, Florida, United States; University of Michigan, Ann Arbor, Michigan, United States; University of Alabama at Birmingham, Birmingham, Alabama, United States; University of Bamberg, Bamberg, Germany

## Abstract

**Objectives:**

Chronic stress has a strong theoretical link to poorer cognitive aging outcomes. Stress from caregiving, especially dementia caregiving, is associated with worse cognition; however, most prior studies have serious methodical limitations. We examined the longitudinal impact of transitioning into caregiving (including dementia caregiving) on cognition, compared to carefully matched non-caregiver controls and possible mediating role of depressive symptoms and perceived stress.

**Methods:**

Participants in the Caregiving Transition Study (CTS) who transitioned into caregiving (n = 251) were compared to sociodemographically and health-matched non-caregiving controls (n = 251). Data included 14 years of repeated assessments including timepoints before and after transitions on global cognition (six-item screener), episodic memory (word list learning, delayed word recall), and verbal fluency (letter and animal fluency).

**Results:**

Compared to non-caregivers, transitioned caregivers performed worse on episodic memory and global cognition after transitions with less decline in word list learning and global cognition post-transitions. All cognitive effects were small (Cohen’s d approximately .2 SD units). In adjusted models for subgroup analyses, dementia caregivers performed worse after transitions than non-dementia caregivers in delayed word recall but better on animal fluency. Increases in depressive symptoms mediated the association between caregiving transitions and episodic memory but not global cognition. Perceived stress did not have a mediating role.

**Discussion:**

Caregiving stress was associated with worse cognition, but effects were domain-specific for memory and global cognition, and short-lived. Dementia vs. non-dementia caregiving also had differential effects on cognition. Future studies should examine whether these small, temporary declines in caregiver cognition improve with caregiver interventions.

